# Mesoporous TiO_2_@g-C_3_N_4_ Nanostructure-Enhanced Photocatalytic Degradation of Tetracycline Under Full-Spectrum Sunlight

**DOI:** 10.3390/molecules29245981

**Published:** 2024-12-18

**Authors:** Lizhe Ma, Zhiyong Fang, Jieli Duan, Jin Li, Kefu Zhu, Yinlong Jiang, Bang Ji, Zhou Yang

**Affiliations:** 1 College of Engineering, South China Agricultural University, Guangzhou 510642, Chinaduanjieli@scau.edu.cn (J.D.);; 2 School of Intelligent Engineering, Shaoguan University, Shaoguan 512005, China; 3 College of Mechanical and Electrical Engineering, Hunan Agricultural University, Changsha 410128, China; 4 School of Mechanical Engineering, Guangdong Ocean University, Zhanjiang 524088, China

**Keywords:** photocatalytic, antibiotic, m-TiO_2_@g-C_3_N_4_, photonic efficiency

## Abstract

TiO_2_ has broad prospects in reducing the safety risks posed by emerging pollutants in water environments. However, the high recombination rate of photogenerated carriers limits the activity and photon utilization efficiency of TiO_2_. In this study, mesoporous TiO_2_ (m-TiO_2_) and ultra-thin g-C_3_N_4_ nanosheets were composited using a hydrothermal method, with the m-TiO_2_ tightly and uniformly wrapped by g-C_3_N_4_. The chemical structure, elemental composition, and optical properties of the heterojunction were analyzed by X-ray diffraction (XRD), X-ray photoelectron spectroscopy (XPS), Fourier transform infrared spectroscopy (FT-IR), and ultraviolet-visible diffuse reflectance spectroscopy (UV-vis-DRS). The activity of the m-TiO_2_@g-C_3_N_4_ was evaluated by the degradation of tetracycline hydrochloride (TCH). Results showed that the heterojunction exhibited significantly enhanced reactivity compared to pure m-TiO_2_ and g-C_3_N_4_, with kinetic rates of TCH being 1.48 and 6.84 times that of pure m-TiO_2_ and g-C_3_N_4_, respectively. The TCH degradation kinetic rate varied from 0.194 min^−1^ to 0.026 min^−1^ and then decreased to 0.015 min^−1^ on the scale of the bandgap and the number of absorbed photons in m-TiO_2_@g-C_3_N_4_. Concurrently, a 10wt% doping amount of g-C_3_N_4_ significantly increased the reaction rate of photogenerated carriers in the system compared to the recombination rate, corresponding to excellent photon efficiency. Reproducibility was evaluated, and a possible degradation mechanism is proposed. This study opens new perspectives for the optimization of catalyst preparation processes aimed at enhancing photon efficiency.

## 1. Introduction

Tetracyclines are a class of broad-spectrum, highly effective, and low-cost antibiotics that inhibit protein synthesis by preventing aminoacyl-tRNA from binding to bacterial ribosomal receptors. As a result, they are widely used in treatment and animal husbandry [1,2,3]. According to a US Food and Drug Administration (US FDA) report for 2021, tetracyclines accounted for 66–67% of total antibiotic consumption [4]. However, limited by the metabolic rate in the body, most of the ingested tetracyclines (~40–90%) will eventually be released into the natural environment [5]. In addition, hospitals, pharmaceutical plants, and sewage treatment plants are also the main sources of release of emerging pollutants such as tetracyclines, which leads to frequent detection in surface water and groundwater, with a detected concentration of ng/L to μg/L [6,7,8]. Furthermore, in water environments, tetracyclines can persist due to their longer half-life [1]. This persistence leads to long-term ecotoxicological effects of tetracyclines on microorganisms and organisms in water ecosystems [9]. Furthermore, tetracyclines can accumulate through the food chain, potentially affecting higher-trophic-level organisms, including humans [10,11]. Therefore, feasible technologies to remove tetracycline drugs from water environments have become crucial to prevent the spread of resistance genes and the development of resistance, given that conventional treatment methods have been ineffective in removing antibiotics.

With this objective, researchers have developed various methods to remove antibiotics from water environments, which can be categorized as biological, physical, and chemical methods [12,13,14]. Among these, photocatalytic oxidation stands out as a powerful technology that harnesses light energy to drive chemical reactions. This method has been extensively studied in such areas as degrading emerging pollutants, reducing CO_2_, splitting water to produce hydrogen, and sterilization [15,16,17,18]. Photocatalysis is also considered to be one of the most promising technologies for removing emerging pollutants due to its low cost, high efficiency, and environmental friendliness. In semiconductor photocatalysts, TiO_2_’s suitable bandgap, non-toxicity, low cost, chemical stability, etc., indicate that it has great application potential. However, it has inherent limitations, such as only absorbing UV light and rapid recombination of photogenerated carriers [19]. Matsuzaki et al. reported that the recombination rate of photogenerated carriers is much higher than the separation rate, with a recombination time of about 100 ps and a diffusion distance greater than 24 nm [19]. In an effort to extend the lifespan of these carriers, researchers have achieved various enhancements of TiO_2_, including metal/non-metal doping, constructing heterojunctions, leveraging defect effects, and introducing co-catalysts [20]. Among these approaches, the formation of heterojunctions has proven to be particularly effective in inhibiting carrier recombination and enhancing carrier mobility.

When it comes to improving the heterojunctions of TiO_2_, the arrangement of band structures and sufficient band-edge potentials are crucial factors. g-C_3_N_4_ is one of the most promising options for constructing TiO_2_ heterojunctions for several reasons: (1) the significant energy difference between the conduction and valence bands (VB) of TiO_2_ and g-C_3_N_4_ promotes the separation of photogenerated carriers; (2) the conjugated π structure facilitates rapid electron transfer across the interface; and (3) the narrow bandgap of g-C_3_N_4_ can act as a sensitizer for TiO_2_, broadening the light absorption range of TiO_2_ [19,21]. Zhang et al. reported that the degradation rate of potassium butyl xanthate using TiO_2_/g-C_3_N_4_ under simulated sunlight was 22 times faster than with pure TiO_2_ [22]. Additionally, under halogen lamp irradiation, the degradation rate of tetracycline by 2D-2D TiO_2_/g-C_3_N_4_ was 1.87 times higher than with pure TiO_2_ [23]. Despite significant progress, previous research has mainly focused on finding the optimal ratio of TiO_2_ to g-C_3_N_4_. In principle, enhancing the effective contact area of TiO_2_ and g-C_3_N_4_ can promote the oxidation activity and durability of the heterojunction. Inspired by the exceptional degradation performance of methylene blue achieved with the traditional core–shell structure TiO_2_@ZnO by Mousa et al., constructing a core–shell structure of mesoporous TiO_2_ (m-TiO_2_) and ultra-thin g-C_3_N_4_ nanosheets could be a promising approach to enhance the oxidation performance of the TiO_2_/g-C_3_N_4_ system [24].

In this study, we prepared m-TiO_2_@g-C_3_N_4_ composite material via a hydrothermal method, with ultra-thin g-C_3_N_4_ nanosheets tightly wrapped around m-TiO_2_. Compared to conventional composite processes, m-TiO_2_ offers a larger surface area for g-C_3_N_4_ interaction to form strong chemical bonds, reducing the distance for e-transfer. Additionally, the physical and chemical properties of m-TiO_2_@g-C_3_N_4_ were examined by XRD, UV-vis DRS, TEM, XPS, and FT-IR. The optimal preparation process of m-TiO_2_@g-C_3_N_4_ was determined through degradation experiments of tetracycline hydrochloride (TCH), and the stability of m-TiO_2_@g-C_3_N_4_ was evaluated. The quantification of the number of photons absorbed by the catalyst was used to analyze the recombination–reaction boundary of photogenerated carriers in the optimized preparation process. Chemical quenching experiments were conducted to better understand the enhanced photocatalytic mechanism proposed for m-TiO_2_@g-C_3_N_4_.

## 2. Results and Discussion

### 2.1. Characterization of the Photocatalyst

#### 2.1.1. XRD

**Figure 1:**
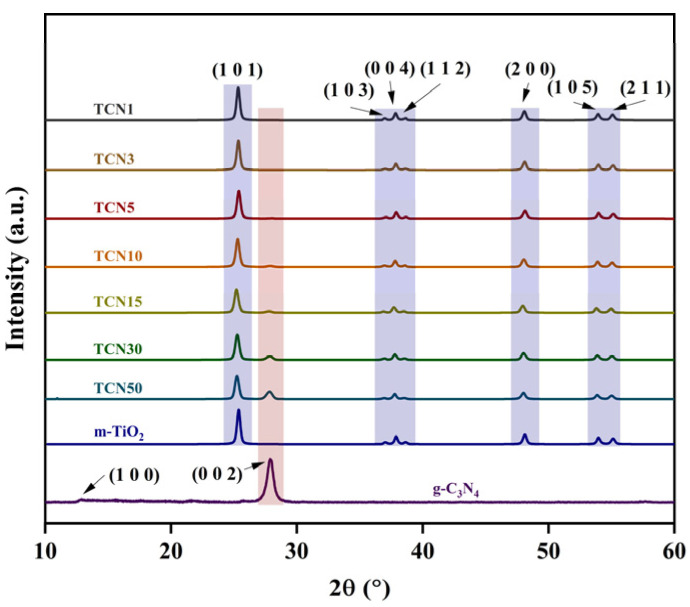
XRD patterns of m-TiO_2_, g-C_3_N_4_, and TCNx.

The crystal phases of the as-synthesized samples were characterized by XRD, and the results are shown in Figure 1. The clear diffraction peaks of m-TiO_2_ at 2θ = 25.2°, 36.8°, 37.7°, 38.4°, 47.9°, 53.8°, and 54.9° are attributable to the (1 0 1), (0 0 4), (2 0 0), (1 0 5), and (2 1 1) planes of anatase TiO_2_, respectively (JCPDF 21-1272) [25,26]. In the g-C_3_N_4_ pattern, two peaks are observed in the 10–60° range, indicating that melamine successfully converts to g-C_3_N_4_. The strong peak at 27.8° corresponds to the (0 0 2) plane and is attributed to the interlayer stacking structure of the aromatic compound, and the weak peak at 12.9° corresponds to the (1 0 0) plane and is assigned to the plane repeat period of tri-s-triazine [27]. All peaks in the m-TiO_2_@g-C_3_N_4_ pattern can be assigned to the characteristic peaks of m-TiO_2_ and g-C_3_N_4_, which indicates that melamine in the precursor is also successfully converted to g-C_3_N_4_ by calcination. It is noteworthy that the intensity of the peak at 26.9° in the TCN1 and TCN3 pattern is very weak, which may be due to the low loading amount of g-C_3_N_4_ in the m-TiO_2_@g-C_3_N_4_.

#### 2.1.2. UV-Vis

**Figure 2:**
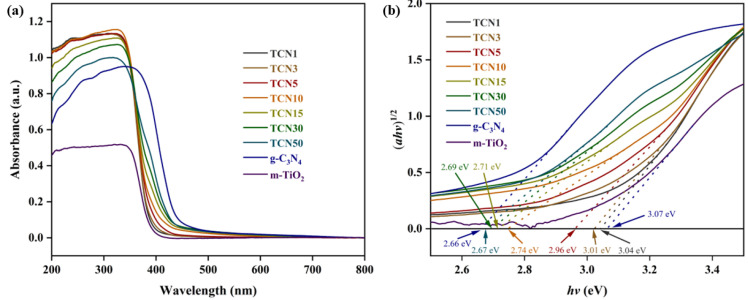
(**a**) UV-vis diffuse reflectance spectroscopy of the samples; (**b**) bandgaps of the samples.

The photophysical properties of m-TiO_2_, g-C_3_N_4_, and TCNx were studied by UV-vis spectroscopy, as shown in Figure 2a. The results showed that all samples have strong absorption in the UV range, which is attributed to the *e*^−^ transition of O 2p electrons to Ti 3d [28]. The bandgaps of all samples were calculated by Equation (1):

(1) ahv=Ahv−Egn2

where *a* is the absorption coefficient; *h* is the Planck constant; *v* is the frequency of light (the ratio of the light speed to wavelength); *A* is a constant; and *E_g_* is the bandgap. The *n* is determined using the sample transition type (*n* = 1 means direct transition, *n* = 4 means indirect transition). Draw *ahv^1/2^*-*hv* and make a tangent at the inflection point of the curve. The results are shown in Figure 2b. The bandgaps of m-TiO_2_ and g-C_3_N_4_ are 3.07 eV and 2.66 eV, respectively. As the content of g-C_3_N_4_ in TCNx increases, the bandgap of TCNx continues to decrease and always falls between that of m-TiO_2_ and g-C_3_N_4_. This is attributed to the electronic coupling effect between m-TiO_2_ and g-C_3_N_4_, which may constitute a charge transfer channel, resulting in the shift of the valence band (VB) and conduction band (CB) positions of TCNx. Among all TCNx, TCN50 has the lowest bandgap (2.67 eV), which represents the minimum energy required for light absorption of TCNx.

#### 2.1.3. TEM Morphological Investigation

**Figure 3:**
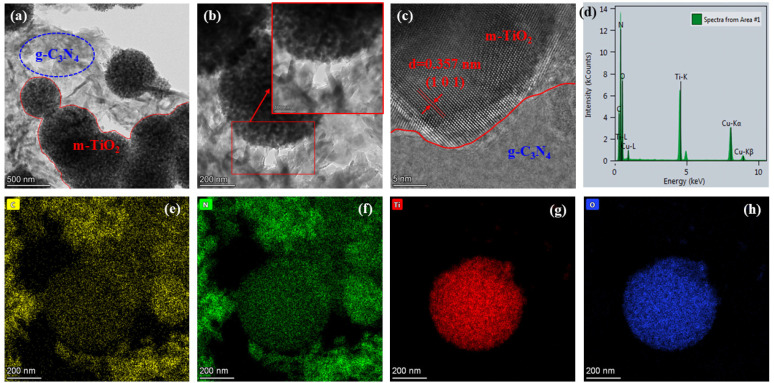
Morphological characterizations of m-TiO_2_@g-C_3_N_4_. (**a**–**c**) TEM images of m-TiO_2_@g-C_3_N_4_; EDX mapping (**d**) of C (**e**), N (**f**), Ti (**g**), and O (**h**).

The morphology structure of m-TiO_2_@g-C_3_N_4_ were studied by HRTEM. Figure 3a,b show that the prepared TiO_2_ is spherical with mesopores on the surface, and the average size of m-TiO_2_ particles is about 488.15–681.01 nm. The surface of m-TiO_2_ particles was covered by well-dispersed g-C_3_N_4_. The different interface edges of m-TiO_2_ and g-C_3_N_4_ are clearly observed in Figure 3c. During the preparation of HRTEM samples, even if the samples were ultrasonically treated, the heterojunction was still strong. Furthermore, the lattice fringes with an interface distance of d = 0.357 nm are also shown, corresponding to the (1 0 1) interface of anatase TiO_2_. The elemental mapping image of the TCN10 sample is further provided, as shown in Figure 3d–h. The presence of Cu-Kα and Cu-Kβ signals indicates that the sample surface was influenced by Cu-Kα radiation, as the samples had undergone XRD and XPS testing prior to HR-TEM analysis. The well-dispersed Ti, O, C, and N elements were observed in the field of view, indicating that the m-TiO_2_ was uniformly distributed in the g-C_3_N_4_ network.

#### 2.1.4. XPS

**Figure 4:**
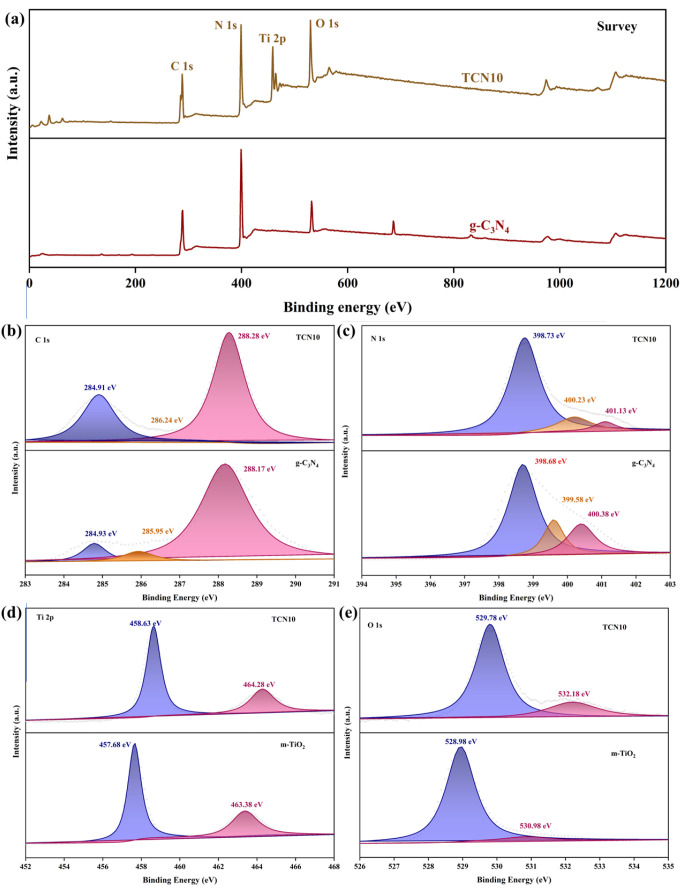
High-resolution XPS spectra of m-TiO_2_@g-C_3_N_4_. (**a**) XPS survey spectra and (**b**) comparison of C 1s and (**c**) comparison of N 1s and (**d**) comparison of Ti 2p and (**e**) comparison of O 1s.

The surface chemical composition and chemical states of m-TiO_2_@g-C_3_N_4_ were analyzed by XPS, and the results are shown in Figure 4. The XPS survey spectrum of g-C_3_N_4_ shows that g-C_3_N_4_ primarily consists C and N elements, with a small presence of O. In comparison, TCN10 clearly demonstrates the coexistence of C, N, Ti, and O elements (Figure 4a), which is consistent with the results of XRD. The C1s of pure CN can be fitted into three peaks at 284.93 eV, 285.95 eV, and 288.17 eV, which are assigned to C-C, C-NH_2_, and N-C=N [29,30] (Figure 4b). Compared with the C 1s peak of pure CN, the C-C peak intensity detected in TCN10 is significantly enhanced, which means: (1) the incorporation of part of the carbon plane; and (2) C-C bonds generated from carbon impurities adsorbed from the surrounding environment [31,32]. The peaks at 399.68, 399.58, and 400.38 eV in g-C_3_N_4_ are assigned to C-N=C, C-N-(-C)-C, and C-N-H, respectively (Figure 4c). Although the positions of the peaks in TCN10 are similar to those in g-C_3_N_4_, the peak areas corresponding to C-N-(-C)-C and C-N-H are significantly reduced, indicating that during the synthesis of TCN10, oxygen partially replaced the combination of uncondensed -NH_2_ and carbon species on the tris-triazine ring [33].

The Ti 2p (Figure 4d) shows two peaks at 458.63 and 464.28 eV, which are attributed to the binding energies of Ti 2p 3/2 and Ti 2p 1/2, respectively. The distance between 2p 3/2 and Ti 2p 1/2 is 5.65 eV, which is a unique structure of Ti^4+^ [34]. The blueshift of the Ti 2p peak in TCN10 indicates the strong interfacial interaction between g-C_3_N_4_ and TiO_2_, which enhances the photogenerated carriers’ separation of the TCN10. The O 1s peaks of CN in Figure 4e are located at 528.98 eV and 530.98 eV, respectively, which are identified as Ti-O-Ti bonds and surface -OH groups. The blueshift of the Ti-O bond in TCN10 suggests that electrons migrated from TiO_2_ to g-C_3_N_4_ [35]. The XRD, TEM, and XPS jointly demonstrate the successful construction of the TCN.

#### 2.1.5. FT-IR

**Figure 5:**
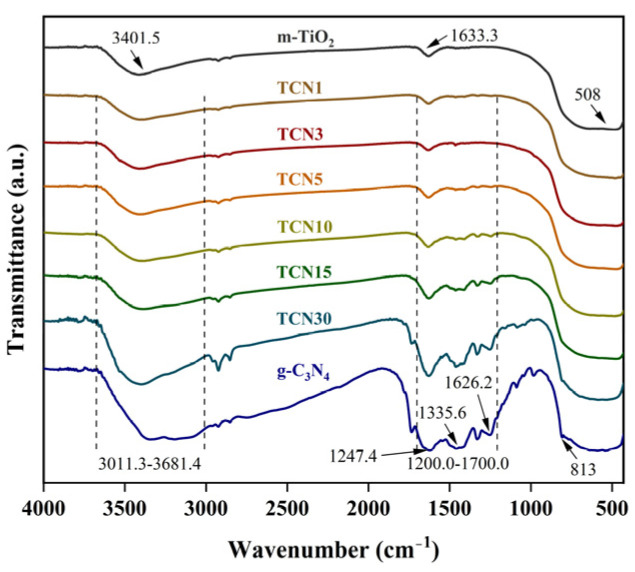
FT-IR spectra of m-TiO_2_, g-C_3_N_4_, and TCNx.

The FT-IR spectra of m-TiO_2_, g-C_3_N_4_ and TCNx are shown in Figure 5. The FT-IR curve trends for TCN samples with different composite ratios show similar changes. For anatase m-TiO_2_, the peak at 508 cm^−1^ is related to the stretching vibration of Ti-O-Ti [34]. The peaks at 1633.3 and 3401.5 cm^−1^ correspond to the bending and stretching vibrations of O-H, which may be related to the adsorption of Ti-OH and H_2_O on the surface of TiO_2_ [36]. For g-C_3_N_4_, the absorption peak at 813 cm^−1^ is due to the bending vibration of the triazine ring unit. The broad peak in the range of 1200.0–1700.0 cm^−1^ belongs to CN heterocycles, including typical sp^2^ C-N (1626.2 cm^−1^) and aromatic sp^3^ C=N (1247.4 cm^−1^, 1335.6 cm^−1^) bonds [37]. The wide absorption band from 3011.3 cm^−1^ to 3681.4 cm^−1^ is caused by the stretching vibrations of O-H groups from H_2_O adsorbed on the g-C_3_N_4_ surface and the terminal NH groups of g-C_3_N_4_ molecules [38]. Moreover, the spectra of all TCNx samples combine characteristic peaks of m-TiO_2_ and g-C_3_N_4_, and the intensity at 3011.3–3681.4 cm^−1^ increases with the increase in CN loading. The results indicate that CN has been successfully loaded onto the surface of m-TiO_2_, and the formation of m-TiO_2_@g-C_3_N_4_ is attributed to the interfacial connection between its components, which is consistent with the characterization results.

### 2.2. Catalytic Performance

**Figure 6:**
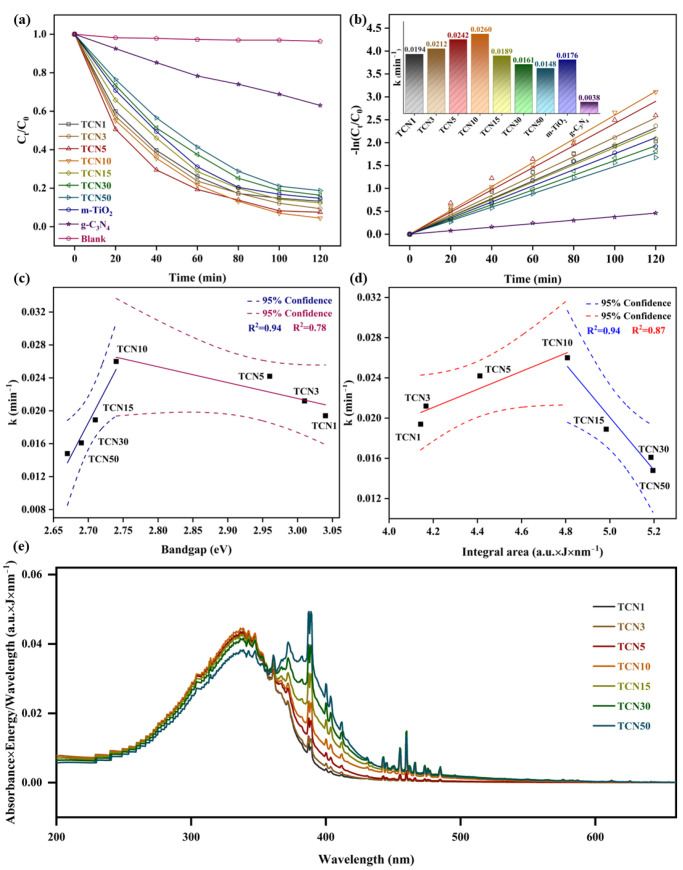
(**a**) Photocatalytic performance of m-TiO_2_, g-C_3_N_4_, and TCNx in removing TCH. (**b**) Degradation kinetics. (**c**) Relationship between bandgap and the degradation kinetic rate. (**d**) Relationship between number of photons absorbed by the catalyst and the degradation kinetic rate. (**e**) Coupling of catalyst and characteristic spectrum of xenon lamp.

The photocatalytic oxidation of TCH by m-TiO_2_, g-C_3_N_4_, and TCNx was studied. Pure g-C_3_N_4_ could remove only 37.01% of TCH after 120 min, due to the hybridization of N 2p and C 2p in the CB of g-C_3_N_4_ accelerating the recombination of photogenerated carriers [19]. Under the same conditions, TCNx demonstrated significantly stronger photocatalytic activity compared to g-C_3_N_4_ and TiO_2_, as shown in Figure 6a. TCN10 showed the best oxidation performance within 120 min. The corresponding TCH degradation rate was 95.53%, while TiO_2_ and CN were 85.27% and 37.01%, respectively. The stable TCH concentration without catalysts indicated that the oxidation activity was driven by the catalyst. To quantitatively assess the kinetics, the pseudo-first-order kinetic rate constants (k) were calculated, as depicted in Figure 6b. The maximum kinetic rate constant *k* of TCN10 was 0.026 min^−1^, which was 1.48 and 6.84 times higher than that of m-TiO_2_ and g-C_3_N_4_, respectively, highlighting the importance of coupling m-TiO_2_ and g-C_3_N_4_ in synergistically enhancing photocatalytic activity. For TCN1, TCN3, TCN5, and TCN10, the *k* values were linearly negatively correlated with the bandgap (Figure 6c), indicating that the heterojunction of the matched electronic band structures between m-TiO_2_ and g-C_3_N_4_ promoted the transfer of photo-induced *e*^−^, thereby improving the photocatalytic performance. However, the *k* values of TCN15, TCN30, and TCN50 were linearly positively correlated with the bandgap, suggesting that increasing the doping amount of g-C_3_N_4_ significantly enhanced the recombination rate of photogenerated charge carriers. The relationship between the number of photons absorbed by the catalyst and the degradation kinetic rate of TCH provided evidence of this, as shown in Figure 6d. In the photocatalytic reaction, the integral area of the spectrum formed by coupling the absorption spectrum of the catalyst and the emission spectrum of the light source was proportional to the number of photons that the catalyst can absorb. The integral area of the coupled spectrum is calculated according to Equation (2):

(2) $$Integral\;area=\int_{\lambda_{min}}^{\lambda_{max}}(C_{Absorbance}\times C_{Emission}∕V_{Wavelength})d\lambda$$

where *C_Absorbance_*, *C_Emission_*, and *V_Wavelength_* are defined as the catalyst absorption spectrum curve, Xenon lamp emission spectrum curve, and their corresponding wavelength values, respectively. *λ_min_* and *λ_max_* are defined as the interval of integration. The integral area of the photocatalytic reaction system (TCNx, Xenon lamp) was calculated using *λ_min_* = 200 nm and *λ_max_* = 660 nm, and the results as shown in Figure 6e. In general, the number of photons absorbed by the catalyst was linearly positively correlated with the degradation kinetic rate of pollutant [19]. However, when the photon energy absorbed by the catalyst is too high, all reaction sites are in a permanent active state and the reaction is completely limited by intrinsic kinetics [39]. An obvious result is that the recombination of photogenerated carriers is aggravated, resulting in a significant inhibition of pollutant degradation. In this study, when the number of photons absorbed by TCN exceeded 4.81 a.u.∙J∙nm^−1^ (corresponding to TCN10), both the degradation kinetic rate and degradation rate of TCH significantly decreased, indicating an excess of photon energy beyond what is required for light saturation of the reaction system. Further, according to the dependency of the number of absorbed photons and the kinetic rate, the photon efficiency of the TCN10–TCH system is optimal.

### 2.3. Energy Consumption

**Figure 7:**
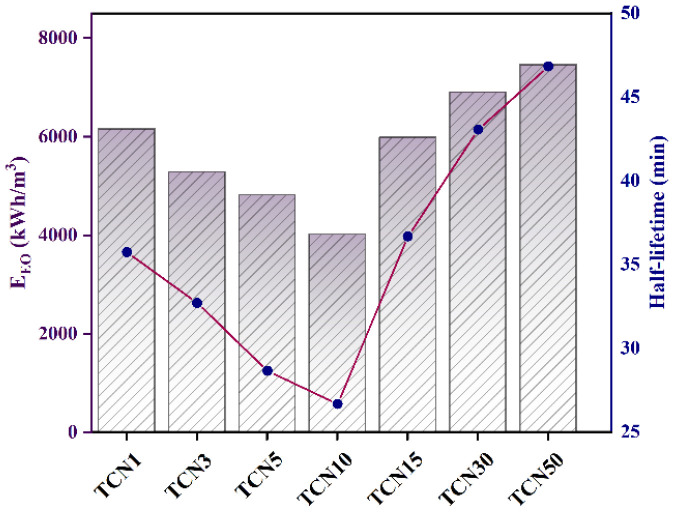
*E_EO_* and half-life in the degradation of TCH by TCNx.

In the process of using xenon light to degrade pollutants in water environment, the consumption of electric energy accounts for an important proportion of the operating cost of the whole process. The electrical energy per order (*E_EO_*) has been widely used in the energy consumption evaluation of various reaction systems. *E_EO_* is defined as the electric energy required to reduce the pollutant concentration of 1 m^3^ of polluted water by 90%, as shown in Equation (3) [40]:

(3) $$E_{EO}=\frac{P\times t\times 1000}{V\times 60\times log(c_{0}/c_{t})}$$

where *P* is the light source power, *V* is the volume of water to be treated, and *C*_0_ and *C_t_* are the initial concentration of water to be treated and the concentration at reaction time *t*, respectively. The results are shown in Figure 7, where the slope represents the electric energy consumption value of TCNx. In the xenon lamp–TCNx system, *E_EO_* varies between 4021 and 7462 kWh/m^3^, and the lowest value occurs in the case of xenon lamp–TCN10. The half-life is defined as the time required for the pollutant concentration in the water environment to decrease from *C*_0_ to 0.5*C*_0_, as shown in Equation (4):

(4) $$t^{0.5}=ln2/k$$

where *k* is the pseudo-first-order kinetic rate constant. The half-life results of the xenon lamp–TCNx system are shown in Figure 7. The half-life and *E_EO_* results are similar, proving that 10 wt% is the optimal ratio of g-C_3_N_4_.

### 2.4. Evaluation of the Lifespan

**Figure 8:**
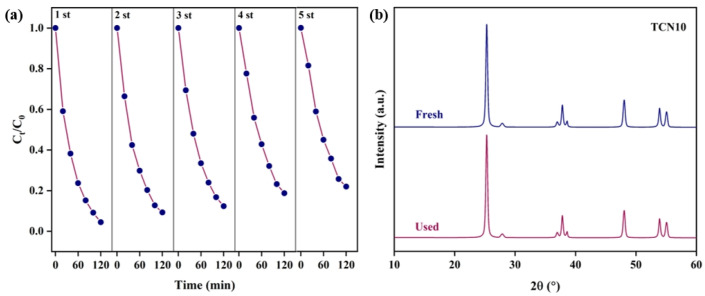
(**a**) Cycling of TCN10 for degradation of the TCH. (**b**) XRD patterns fresh and after TCN recycling.

A stable photocatalyst is very important to improve the economy of the photocatalytic degradation process. Catalyst deactivation, reoxidation of reduction products, and poisoning effects may result in the catalyst losing stability under long-term irradiation. Lifespan testing of TCN photocatalytic degradation of TCH was carried out under xenon lamp irradiation, as shown in Figure 8a. After each round of degradation, TCN was washed with deionized water and dried for the next round of photocatalytic testing. Figure 8a shows that the degradation rates of TCH for the five cycles were 95.53%, 90.72%, 89.66%, 88.26%, and 87.07%, indicating that TCN has stable oxidation activity in the degradation of TCH. In addition, there was no significant change in the XRD pattern (Figure 8b) between the fresh TCN10 photocatalyst and the TCN10 after photocatalytic reaction.

### 2.5. Mechanism of the System

**Figure 9:**
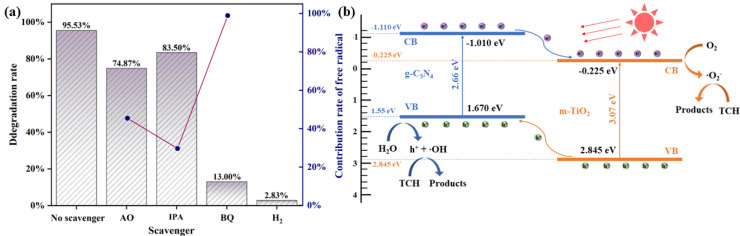
(**a**) Effect of free radical scavengers on TCN degradation TCH. (**b**) Possible photocatalytic mechanism of degradation of TCH by TCN on simulated sunlight.

To distinguish the contribution of active species to TCH degradation, free radical scavenging experiments were performed, as shown in Figure 9a. Ammonium oxalate (AO), isopropyl alcohol (IPA), and 1,4-benzoquinone (BQ) were utilized to scavenge *h*^+^, ‧OH, and ·O_2_^−^, respectively. The findings revealed that the degradation percentage of TCH decreased from 95.53% (no scavenger) to 74.87% (AO), 83.50% (IPA), and 13.00% (BQ). This indicated a weak correlation between *h*^+^, ·OH and TCH degradation. ·O_2_^−^ was a primary factor in the photocatalytic degradation process, and its contribution rate was calculated by Equation (5):

(5) $$R_{RS}=\frac{k_{RS}}{k}\approx \frac{\left(k-k_{i}\right)}{k}$$

where *R_RS_* is the fractional contribution of a specific active species to the kinetic rate constant. *k* (min^−1^) is the apparent rate constant of the reaction without quenching agents, and *k_i_* is the apparent rate constant for TCH degradation of the specific reactive species. The result showed that the contribution rate of ·O_2_^−^ was approximately 98.80%. Furthermore, when N_2_ was blown into the reaction solution, the degradation rate of TCH was sharply inhibited, indicating that O_2_ is an important co-substrate for TCN to degrade TCH, and the reduction and activation of O_2_ to ·O_2_^−^ by photogenerated *e*^−^, which is the dominant step in the degradation of TCH.

(6) $${m-TiO}_{2}@{g-C}_{3}N_{4}+hv\to {m-TiO}_{2}@{g-C}_{3}N_{4}(e^{-}+h^{+})$$

(7) $${m-TiO}_{2}@{g-C}_{3}N_{4}(e^{-}+h^{+})\to {m-TiO}_{2}(e^{-})+{g-C}_{3}N_{4}(h^{+})$$

(8) $$e^{-}+O_{2}\to \cdot O_{2}^{-}$$

(9) $$h^{+}+H_{2}O\to \cdot OH$$

(10) $$\cdot O_{2}^{-}/\cdot OH/h^{+}+TCH\to {CO}_{2}+H_{2}O+intermediate\;product$$

Based on the above results, the photocatalytic mechanism of the TCN heterojunction under UV-vis light may be obtained, as shown in Figure 9b. The VB and CB positions of m-TiO_2_ and g-C_3_N_4_ are calculated based on the absolute electronegativity, as shown in Equations (11) and (12):

(11) $$E_{VB}=X-E_{e}+0.5E_{g}$$

(12) $$E_{CB}=E_{VB}-E_{g}$$

where *X* is the electronegativity of catalytic, *E_e_* is the energy of free electronic at hydrogen scale, which is usually 4.5 eV. The electronegativity of TiO_2_ and g-C_3_N_4_ were determined to be 5.81 eV and 4.72 eV, respectively [41,42]. The *E_cb_* and *E_vb_* of TiO2 were calculated to be −0.225 eV and 2.845 eV. Similarly, the of g-C_3_N_4_ was calculated to be −1.11 eV and 1.55 eV, respectively. The m-TiO_2_ and g-C_3_N_4_ formed a staggered band, and TCN was excited under UV-vis light irradiation (Equation (6)). The *e*^−^ produced by g-C_3_N_4_ tended to converge to the CB of m-TiO_2_ (Equation (7)). The enriched *e*^−^ in the VB of m-TiO_2_ reacted with the dissolved O_2_ adsorbed on the surface of TCN to form a strong oxidizing ·O_2_^−^ (Equation (8)). The direction of transport of *h*^+^ and *e*^−^ between m-TiO_2_ and g-C_3_N_4_ was opposite, and a small number of *h*^+^ enriched in the VB of g-C_3_N_4_ reacted with H_2_O to form ‧OH (Equation (9)). Subsequently, TCH molecules were decomposed into CO_2_ and H_2_O by *h*^+^, ·OH, and ·O_2_^−^ (Equation (10)). It should be noted that the degradation of TCH by *h*^+^, ·OH, and ·O_2_^−^ does not have to occur in a set order or at the same time, due to TCN having the ability to store excess *h*^+^ for a short period [39]. According to previous reports, in the TCH–TiO_2_ system, the surface of TiO_2_ can form a complex that can be excited under visible-light irradiation, and the released *e*^−^ converged in the CB of TiO_2_ and promoted the degradation of TCH by TiO_2_ [43,44]. However, the degradation of TCH under UV-vis light irradiation is dominated by TCN itself, and the enhancement of the degradation effect of TCH by the complexes is almost negligible. In summary, the formation of the TCN heterojunction can effectively improve the separation of photogenerated *e*^−^–*h*^+^ pairs, thereby improving the photocatalytic performance.

## 3. Experimental

### 3.1. Chemicals and Material Preparation

#### 3.1.1. Chemicals

TCH, melamine, 1-hexadecylamine (HDA), absolute ethanol, titanium isopropoxide, and potassium chloride (KCl) were purchased from Shanghai Macklin Biochemical Co., Ltd. (Shanghai, China) *p*-Benzoquinone (BQ, C_6_H_4_O_2_, ≥99.5%), isopropyl alcohol (IPA, C_3_H_8_O, ≥99.5%), and sodium oxalate (AO, C_2_Na_2_O_4_, 99.5%) were purchased from Aladdin Reagent Co., Ltd. (Shanghai, China). All chemicals were of analytical purity and all solutions were prepared in DI water. All other materials used in this study were analytical grade and used without further treatment. DI water was prepared in the laboratory and used throughout the experiments.

#### 3.1.2. Preparation of m-TiO_2_

In a typical process for preparing m-TiO_2_, 1.9 g of HDA was dissolved in 200 mL of anhydrous ethanol. Then, 1.6 mL of 0.1 M KCl solution was slowly added to the solution, followed by the introduction of 5 mL of titanium isopropoxide while stirring, resulting in a milky-white suspension. The mixture was left at room temperature for 24 h to allow for complete reaction. After filtration, centrifugation, and drying, titanium salt powder was obtained. To prepare m-TiO_2_, 1.0 g of the powder was mixed with 40 mL of anhydrous ethanol and 20 mL of DI water, and the mixture was shaken for 1 h. The solution was then transferred to a 100 mL Teflon-lined stainless-steel autoclave and hydrothermally treated at 160 °C for 20 h. Following filtration, centrifugation, and drying, a white powder was obtained. Finally, the powder was calcined at 500 °C in a muffle furnace (heating rate: 5 °C/min) for 2 h to yield m-TiO_2_.

#### 3.1.3. Preparation of g-C_3_N_4_

g-C_3_N_4_ was synthesized using the thermal polymerization method. Initially, melamine was placed in a covered crucible, tightly wrapped in aluminum foil, and heated in a muffle furnace at 550 °C for 4 h. After cooling and grinding, g-C_3_N_4_ was obtained. Next, 1 g of g-C_3_N_4_ was mixed with 100 mL of 37% HCl and stirred for 12 h. The resulting mixture was then filtered, washed to neutrality, and dried at 80 °C overnight to yield ultra-thin carbon nitride nanosheets.

#### 3.1.4. Preparation of m-TiO_2_@g-C_3_N_4_

The m-TiO_2_@g-C_3_N_4_ composite material was prepared using a hydrothermal synthesis method. Initially, an appropriate quantity of m-TiO_2_ was dispersed in DI water, subjected to 15 min of ultrasonic treatment, and stirred for 15 min. Subsequently, the necessary amount of g-C_3_N_4_ was added, followed by 30 min of continuous ultrasonic treatment and 20 min of stirring. The resulting suspension was then transferred to a high-pressure reaction vessel and maintained at 180 °C for 4 h. After cooling, filtration, drying, and grinding, the m-TiO_2_@g-C_3_N_4_ composite material was obtained. Since the mass ratio of g-C_3_N_4_ in the m-TiO_2_@g-C_3_N_4_ composite material was 1, 3, 5, 10, 15, 30, and 50 wt%, the synthesized photocatalysts are denoted as TCN1, TCN3, TCN5, TCN10, TCN15, TCN30, and TCN50, respectively.

### 3.2. Characterization

The powder X-ray diffraction (XRD) patterns were recorded using a SmartLab diffractometer (Rigaku, Tokyo, Japan) with Cu-Kα radiation. The UV-vis diffusion reflectance spectra were obtained directly using a UV-vis spectrophotometer (Shimadzu UV-2600, Kyoto, Japan) with BaSO_4_ as a reference. X-ray photoelectron spectroscopy (XPS) was conducted using an ESCALAB 250XI (Thermo Fisher Scientific, Waltham, MA, USA) with an Al Kα X-ray source (*hν* = 1486.8 eV). The FT-IR spectra were recorded on a VERTEX 70 (BRUKER, Ettlingen, Germany) with an attenuated total reflection setup. Surface morphology and dispersibility of the samples were recorded by high-resolution transmission electron microscopy (HR-TEM, FEI Talos F200i, Thermo Fisher Scientific, Hillsboro, OR, USA). The HR-TEM test samples were obtained from the recycled XRD and XPS test samples.

### 3.3. Photocatalytic Experiments

**Figure 10:**
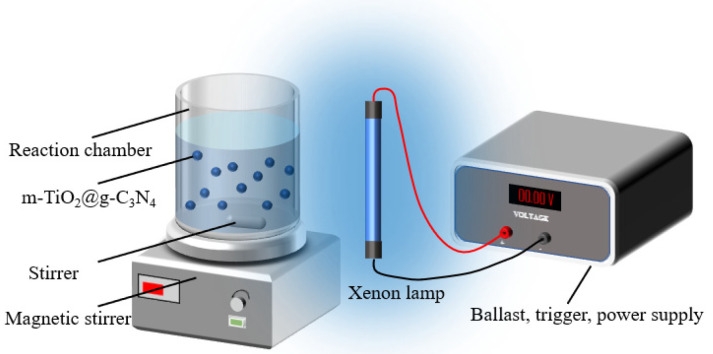
Detailed schematic diagram of the quartz reactor.

The setup of the photocatalytic reaction system is shown in Figure 10. Briefly, the TCH photocatalytic degradation experiments were conducted in a 100 mL quartz reactor at room temperature. A 500 W xenon lamp (Shanghai Jiguang Special Lighting Electric Appliance Factory, Shanghai, China) was used as the visible light source. The distance between the light source and the quartz tube was about 50 cm to make the light intensity uniform and isolate the heat of the light source. Before the reaction, 20 mg of catalyst was dispersed in 80 mL of TCH solution (20 mg/L) and stirred for 30 min in a dark environment to achieve adsorption equilibrium. The time interval for each sampling was 20 min. The catalytic were filtered through a PTFE filter (pore size 0.45 μm, Tianjin Jinteng Experiment Equipment Co., Ltd., Tianjin, China) and the absorbance of TCH at 356 nm was measured using a UV-vis spectrophotometer. To identify the dominant reactive species in TC removal, IPA, AO, and *p*-BQ were used as scavengers for ·OH, *h*^+^, and ·O_2_^−^, respectively, at a concentration of 0.1 mM.

## 4. Conclusions

In this study, we successfully prepared a m-TiO_2_@g-C_3_N_4_ heterojunction photocatalyst through a hydrothermal method. UV-vis DRS, XRD, and XPS confirmed the formation of m-TiO_2_@g-C_3_N_4_, and TEM confirmed that ultra-thin g-C_3_N_4_ nanosheets were uniformly wrapped around m-TiO_2_. Under xenon lamp irradiation, the degradation kinetic rate of TCH increased from 0.194 min^−1^ to 0.026 min^−1^ and then decreased to 0.015 min^−1^ on the scale of the bandgap and the number of photons absorbed by m-TiO_2_@g-C_3_N_4_. TCN10 exhibited the best catalytic activity, with a TCH degradation rate of 95.53%, and the kinetic rate was 1.48 times and 6.84 times that of pure m-TiO_2_ and g g-C_3_N_4_, respectively. The reaction rate of photogenerated carriers in the TCN10–TCH reaction system was greater than the recombination rate, and TCN10 corresponded to excellent photon efficiency. Reproducibility assessment showed that the TCH degradation rate remained above 85% throughout five consecutive experiments. In addition, a possible mechanism for the degradation of TCH by m-TiO_2_@g-C_3_N_4_ was proposed, which may open up new perspectives for the optimization of heterojunction preparation processes.

## Data Availability

Data are contained within the article.
